# Identification of Antimicrobial Resistance Patterns, Type VI Secretion System, and Virulence Factors in Clinical Isolates of *Klebsiella pneumoniae*: A Cross-Sectional Study

**DOI:** 10.1155/ijm/9634257

**Published:** 2025-08-22

**Authors:** Mohammad Hossein Haddadi, Elahe Taki, Abbas Maleki, Jamshid Mashhadi, Fatemeh Shahi, Maryam Koupaei, Nourkhoda Sadeghifard, Reza Faraji, Melika Moradi, Hassan Valadbeigi, Saeed Khoshnood

**Affiliations:** ^1^Clinical Microbiology Research Center, Ilam University of Medical Sciences, Ilam, Iran; ^2^Department of Microbiology, School of Medicine, Kermanshah University of Medical Sciences, Kermanshah, Iran; ^3^Shoushtar Faculty of Medical Sciences, Shoushtar, Iran; ^4^Infectious and Tropical Diseases Research Center, Health Research Institute, Ahvaz Jundishapur University of Medical Sciences, Ahvaz, Iran; ^5^Department of Microbiology and Immunology, School of Medicine, Kashan University of Medical Sciences, Kashan, Iran; ^6^Tuberculosis and Lung Diseases Research Center, Ilam University of Medical Sciences, Ilam, Iran; ^7^Department of Microbiology, School of Medicine, Ahvaz Jundishapur University of Medical Sciences, Ahvaz, Iran

**Keywords:** carbapenem resistance, hypervirulent *K. pneumoniae*, multidrug resistance, Type VI secretion system, virulence factors

## Abstract

**Background:** *Klebsiella pneumoniae* is an opportunistic pathogen linked to a range of severe infections. It is classified into classic *K. pneumoniae* (cKp) and hypervirulent *K. pneumoniae* (hvKp) forms, with hvKp showing enhanced virulence. This study examines Type VI secretion system (T6SS) presence and virulence factors in cKp and hvKp strains while assessing antimicrobial resistance.

**Methods:** Eighty-three *K. pneumoniae* isolates were collected from hospitalized patients in Ilam, Iran, between June and December 2023. Antimicrobial susceptibility testing was conducted using the disk diffusion method. Hypervirulent *K. pneumoniae* isolates were identified by string test, tellurite resistance, and presence of *peg-344*, *iucA*, and *iutA* using polymerase chain reaction (PCR). Furthermore, PCR detected virulence, *β*-lactamases, and T6SS genes.

**Results:** Of 83 isolates, 16.87% and 83.13% were hvKp and cKp, respectively. All isolates were multidrug-resistant (MDR), with 18.07% exhibiting carbapenem resistance. The pattern of antibiotic resistance in hvKp and cKp is not significantly different, except for ciprofloxacin, tetracycline, and azithromycin. Also, there was no significant difference between T6SS-positive and T6SS-negative isolates except for ciprofloxacin. T6SS presence did not significantly correlate with virulence or resistance genes, except for ciprofloxacin resistance (*p* = 0.0219).

**Conclusion:** Surveillance, infection control, and targeted therapies are essential to combat hypervirulent and drug-resistant *K. pneumoniae*.

## 1. Introduction

*Klebsiella pneumonia (K. pneumonia)* is an opportunistic pathogen capable of causing pneumonia, urinary tract infections (UTI), and bacteremia. The highest-risk populations include neonates, elderly individuals, immunocompromised patients, and those frequently exposed to healthcare settings [1, 2]. The pathogenicity of *K. pneumoniae* is attributed to multiple virulence factors, such as capsules, lipopolysaccharides, exopolysaccharides (associated with mucoviscosity), iron uptake systems, and adhesins [3, 4]. Based on virulence profiles, *K. pneumoniae* is broadly classified into classic *K. pneumoniae* (cKp) and hypervirulent (hv) *K. pneumoniae* (hvKp) [5]. hvKp strains are associated with severe community-acquired infections, such as pyogenic liver abscesses, osteomyelitis, and endophthalmitis, typically affecting younger and healthier individuals. Their heightened pathogenicity is primarily due to the overproduction of capsular polysaccharides and siderophores [6, 7]. Additionally, hvKp strains exhibit hypermucoviscous (HMV) because of excessive capsule polysaccharide synthesis [7]. On the other hand, cKp strains are predominantly linked to hospital-acquired infections, such as UTI, pneumonia, meningitis, and endophthalmitis. Several methods have been employed to detect hvKp isolates, including phenotypic assays such as the string test, serum killing assay, mouse infection models, and *Galleria mellonella* infection models. Genetic detection of virulence-associated genes increases the accuracy and sensitivity of identification. Notably, *iucA*, *iutA*, and *peg-344* have been established as the most reliable genetic diagnostic markers. Additionally, key virulence factors, including the regulator of mucoid phenotype A (*rmpA*) and mucoviscosity-associated gene A (*magA*), have been implicated in the HMV phenotype [8]. The *rmpA* gene, located on a 180-kilobase plasmid, acts as a positive regulator of extracapsular polysaccharide synthesis. Furthermore, *mrkD* and *fimH* encode Type 3 and Type 1 fimbriae, respectively, facilitating host cell adhesion. The *iroB* gene, involved in iron acquisition, encodes a protein essential for synthesis of a siderophore virulence factor, which is known as aerobactin [9]. *K. pneumoniae* exhibits intrinsic resistance to *β*-lactam antibiotics, and increasing resistance to carbapenems exacerbates the risk of severe disseminated infections following broad-spectrum antibiotic treatment. Although most hvKp strains remain antibiotic-sensitive, emerging virulent and carbapenem-resistant (CR) strains pose a growing threat. These strains often harbor virulence factors on large plasmids and acquire resistance through mobile genetic elements (MGEs) [7, 10]. The hv phenotype is conferred by genetic determinants located on a large virulence plasmid and possibly integrative conjugative elements. Similar to cKp, hvKp strains are progressively developing antimicrobial resistance through the acquisition of mobile resistance genes [11]. Multidrug-resistant (MDR) hvKp (MDR-hvKp) and carbapenem-resistant hvKp (CR-hvKp) are increasingly recognized worldwide [12], with associated mortality rates ranging from 40% to 50%. Moreover, resistance to last-resort antibiotics, such as colistin, is emerging among CR *K. pneumoniae* strains, with some isolates demonstrating pan-resistance [13]. Like many Gram-negative bacteria, *K. pneumoniae* possesses a Type VI secretion system (T6SS), which plays a crucial role in microbial communication within host environments [14]. The T6SS facilitates the secretion of hemolysin coregulated protein (Hcp) and valine-glycine repeat protein G (VgrG), which contribute to cytotoxic effects and require a cluster of intracellular multiplication protein F (IcmF)-related genes [15]. In *K. pneumoniae*, T6SS mediates bacterial competition, host cell invasion, Type-1 fimbriae expression, and in vivo colonization [5]. Some studies suggest that T6SS-positive strains exhibit enhanced drug resistance and virulence potential [16, 17]. Given these findings, this study is aimed at investigating T6SS and other virulence factors in both cKp and hvKp strains and conduct a comparative analysis between the two.

## 2. Materials and Methods

### 2.1. Bacterial Isolates and Identification

A total of 83 *K. pneumoniae* isolates were obtained from culture-positive patients admitted to Razi Hospital in Ilam, Southwestern Iran, between June and December 2023. These clinical isolates were derived from various specimens, including urine, tracheal aspirates, cerebrospinal fluid (CSF), pleural fluid, urinary catheters, and blood samples. All specimens were cultured aseptically on 5% sheep blood agar and MacConkey agar plates (Merck, Germany) and incubated aerobically at 37°C for 18–24 h. *K. pneumoniae* isolates were identified using Gram staining and a series of biochemical tests, including methyl red (MR), Voges–Proskauer (VP), citrate utilization, lysine iron agar (LIA), triple sugar iron (TSI), sulfide–indole–motility (SIM), and urea hydrolysis. *K. pneumoniae* ATCC 700603 was used as the reference control strain. One confirmed *K. pneumoniae* isolate per sample was selected and stored in nutrient broth (Merck) supplemented with 30% sterile glycerol at −70°C. Ethical approval for this study was obtained from the Ethical Committee of Ilam University of Medical Sciences, in compliance with the Declaration of Helsinki (Ethical Code: IIR.MEDILAM.REC.1401.152). Written informed consent was obtained from all participants prior to sample collection.

### 2.2. Phenotypic and Molecular Identification of HvKp

The HMV phenotype was assessed using the string test, where a positive result was defined as the formation of a viscous string ≥ 5 mm when a bacterial loop was used to stretch a colony grown on blood agar [11]. Tellurite resistance was evaluated using a tellurite-supplemented agar medium. Briefly, 0.1 g of potassium tellurite powder was dissolved in 10 mL of sterile distilled water and filtered through a 0.45-*μ*m membrane. Subsequently, 300 *μ*L of the filtered solution was mixed with 100 mL of autoclaved Mueller–Hinton agar (MHA) cooled to 45°C–50°C. The medium was then poured into sterile plates. After overnight incubation at 37°C, colonies exhibiting a black appearance were considered presumptive hv strains [18]. Isolates that developed black colonies on this medium were classified as presumptive hv strains. All tellurite-resistant *K. pneumoniae* isolates were screened for the presence of *peg-344*, *iucA* (aerobactin synthesis gene), and *iutA* (aerobactin receptor gene). Isolates positive for any of these genes were classified as hvKp [1].

### 2.3. Antimicrobial Susceptibility Testing

Antimicrobial susceptibility testing was performed using the disk diffusion method on MHA (Merck) in accordance with Clinical and Laboratory Standards Institute (CLSI) guidelines. The antibiotics tested included ceftriaxone (CRO; 30 *μ*g), cefotaxime (CTX; 30 *μ*g), cefepime (CFP; 30 *μ*g), chloramphenicol (CHO; 10 *μ*g), tetracycline (TET; 30 *μ*g), imipenem (IMI; 10 *μ*g), meropenem (MEM; 10 *μ*g), amikacin (AMK; 30 *μ*g), gentamicin (GEN; 10 *μ*g), azithromycin (AZM; 15 *μ*g), trimethoprim/sulfamethoxazole (SXT; 1.25/23.75 *μ*g), and ciprofloxacin (CIP; 5 *μ*g). Bacterial suspensions were standardized to a 0.5 McFarland turbidity and inoculated onto MHA plates. Following incubation at 37°C for 18–24 h, inhibition zone diameters were measured. *Escherichia coli* (*E. coli*) ATCC 25922 was used as the quality control strain, and MDR was defined as nonsusceptibility to at least one agent in three or more antimicrobial categories.

### 2.4. Detection of Virulence, *β*-Lactamases, and T6SS Genes

Genomic DNA was extracted from pure *K. pneumoniae* colonies using the boiling method [19], wherein bacterial colonies were boiled in sterile distilled water for 10 min, frozen at −20°C for 10 min, and centrifuged at 13,000 rpm for 10 min, with the resulting supernatant serving as the DNA template. PCR amplification targeted virulence genes (*mrkD*, *iroB*, *peg-344*, *iucA*, *iutA*, and *rmpA*), *β*-lactamase genes (*bla*_KPC-1_, *bla*_OXA-48_, *bla*_NDM-1_, and *bla*_VIM_), and T6SS genes (*hcp*, *vgrG*, and *icmF*). Primer sequences that were used in this study are shown in Table 1.

### 2.5. Statistical Analysis

Descriptive statistics were analyzed using Microsoft Excel and SPSS Version 22 (IBM Corporation, Armonk, New York, United States). Categorical variables were compared using the chi-square test or Fisher's exact test, as appropriate. A *p* value of ≤ 0.05 was considered statistically significant. Results are presented as relative frequencies.

## 3. Results

### 3.1. Study Population and Bacterial Isolates

In this study, a total of 83 clinical *K. pneumoniae* isolates were obtained from various clinical specimens. The distribution of isolates by sample type is presented in Table 2. These isolates were collected from 49 (59.03%) male and 34 (40.96%) female hospitalized patients, with a mean age of 43.4 years (range: 9–71 years). The majority of *K. pneumoniae* isolates were recovered from urine (36; 43.37%), tracheal aspirates (21; 27.63%), and urinary catheters (11; 13.25%). Other sources included blood (9; 10.84%), pleural fluid (5; 6.57%), and CSF (1; 1.31%). The isolates originated from the following hospital wards: infectious diseases (15; 18.07%), pediatrics (5; 6.02%), surgery (8; 10.52%), nephrology (6; 7.22%), neurology (5; 6.57%), emergency (15; 18.07%), and intensive care unit (ICU) (29; 38.15%).

### 3.2. Phenotypic and Molecular Identification of hvKp

Among the 83 isolates analyzed, 31.32% (*n* = 26) exhibited growth on MHA supplemented with tellurite. Additionally, 24.09% (*n* = 20) demonstrated a positive string test, indicative of the HMV phenotype. Molecular analysis revealed hvKp-associated markers (*iucA*, *iutA*, or *peg-344*) in 44.57% (*n* = 37) of all isolates (*iucA*: 15, *iutA*: 8, and *peg-344*: 14). Among tellurite-resistant isolates, 53.84% (*n* = 14/26) harbored these markers (*iucA*: 6, *iutA*: 3, *peg-344*: 6). Furthermore, 21.42% (*n* = 3/14) of hvKp isolates tested positive in the string test. Of the total isolates, 57 did not grow on tellurite-supplemented MHA, while 17 cKp isolates exhibited a positive string test (hvKp isolates: ID 9, 14, 17, 23, 25, 43, 46, 58, 60, 66, 68, 69, 78, and 82). The majority of hvKp isolates were obtained from urine (*n* = 9) and blood (*n* = 3) samples.

### 3.3. Antimicrobial Susceptibility Testing

Antimicrobial susceptibility testing demonstrated that amikacin (78.31% sensitivity, 65/83) and imipenem (84.33% sensitivity, 70/83) were the most effective antibiotics, whereas cotrimoxazole and cephalosporins exhibited the lowest efficacy (Table 3). Resistance rates to other antibiotics were as follows: chloramphenicol (53%), tetracycline (38.55%), meropenem (18.07%), gentamicin (32.53%), ciprofloxacin (56.62%), and azithromycin (54.21%). Among aminoglycosides and carbapenems, gentamicin and meropenem showed the least activity. In hvKp isolates, amikacin, tetracycline, and carbapenems were the most effective, with susceptibility rates exceeding 80%. All *K. pneumoniae* isolates were classified as MDR. The CR rate was 18.07% (*n* = 15), comprising 2.4% (*n* = 2/83) hvKp and 15.66% (*n* = 13/83) cKp isolates. Specifically, 18.84% (*n* = 13/69) of cKp and 14.28% (*n* = 2/14) of hvKp isolates were CR-cKp and CR-hvKp, respectively. Comparative analysis of antibiotic susceptibility between hvKp and cKp isolates revealed no significant differences, except for ciprofloxacin, tetracycline, and azithromycin (*p* = 0.0347, 0.0116, 0.0116, respectively, Table 4). Additionally, no significant differences were observed between T6SS-positive and -negative isolates, except for ciprofloxacin (*p* = 0.0219; Table 5).

### 3.4. Detection of Virulence, Antimicrobial Resistance, and T6SS Genes

The distribution of virulence and resistance genes among T6SS-positive, T6SS-negative, hvKp, and cKp isolates is summarized in Tables 6 and 7. Among string test-positive isolates, nine carried one or two virulence genes, irrespective of tellurite resistance. The most prevalent virulence gene was *iucA* (18.07%, *n* = 15), followed by *peg-344* (16.86%, *n* = 27) and *rmpA* (14.45%, *n* = 12). Among antibiotic resistance genes, *bla*_KPC-1_ (*n* = 25) and *bla*_OXA-48_ (*n* = 14) were the most abundant. The *vgrG* was the most frequently detected gene at 15 (18.07%) followed by *icmF* 11 (13.25%), and *rmpA* 8 (9.63%). There was no significant difference between virulence and drug resistance genes between T6SS-positive and T6SS-negative and hvKp and cKp isolates (Tables 6 and 7).

## 4. Discussion

The primary reservoir of *K. pneumoniae* is the human intestine, which can become contaminated through exposure to environmental sources such as soil, vegetation, surfaces, and water [1]. Infections caused by hvKp and cKp exhibit distinct epidemiological and clinical characteristics: hvKp is more prevalent in community settings, whereas cKp is predominantly associated with hospital-acquired infections. Additionally, hvKp infections occur across all age groups, including healthy individuals, whereas cKp primarily affects the elderly and immunocompromised patients. Unlike cKp, which shows no ethnic specificity, hvKp exhibits a higher incidence in Asian, Pacific Islander, and Hispanic populations. Clinically, hvKp infections often involve multiple sites, leading to rare conditions such as meningitis and brain abscesses, whereas cKp infections are typically localized to the abdomen, soft tissues, and urinary catheters [11]. The objectives of this study were to (i) assess antibacterial resistance in collected strains, (ii) detect the presence of the T6SS, (iii) investigate the association between T6SS and virulence factor genes, and (iv) examine the relationship between T6SS and antibiotic resistance genes. hvKp emerges when cKp acquires virulence determinants, often encoded on a large virulence plasmid, exhibiting heightened pathogenicity and frequently causing infections in immunocompetent individuals, with notably high prevalence in Asia and the Pacific [11].

Key virulence-associated genes include *iucA*, which encodes aerobactin synthesis and is located on plasmid pLVPK [18, 25]; *iutA,* also located on pLVPK [18, 25]; and *peg-344*, a metabolic transporter gene with an undetermined function [26]. Five biomarkers, *peg344*, *iroB*, *iucA*, *rmpA*, and *rmpA,* along with siderophore production (≥ 30 *μ*g/mL), serve as reliable indicators of hvKp. While *peg344* encodes a transmembrane transporter, its role in virulence remains unclear. Notably, *peg344* is present in both MDR-hvKp strains (i and ii) and facilitates rapid hvKp detection. Recent advances include the development of loop-mediated isothermal amplification (LAMP) for hvKp identification [22, 27, 28]. In this study, tellurite resistance and the presence of *iucA*, *iutA*, and *peg344* were used to identify hvKp. Phenotypic analysis indicated a prevalence of 53.84%, whereas genotypic methods yielded 44.57%. The discrepancy suggests that PCR-based detection offers higher diagnostic accuracy, confirming that not all tellurite-resistant strains necessarily harbor hvKp markers.

Following four decades of hvKp strains exhibiting high antibiotic susceptibility, there has been a concerning rise in antibiotic resistance among these isolates [1]. MGEs play a critical role in resistance development [11]. HvKp acquires resistance to antibiotics in several ways. Conjugative plasmids containing resistance genes are one of the mechanisms for acquiring resistance genes [29], integrative and conjugative elements (ICEs) inserted into chromosomes or virulence plasmids [30, 31], and chromosomal mutations, particularly in outer membrane protein genes [31]. Notably, resistance acquisition in hvKp progresses more slowly than in cKp, potentially due to capsule overexpression, plasmid incompatibility, and CRISPR-Cas systems [32]. The emergence of MDR and extensively drug-resistant (XDR) cKp strains acquiring virulence plasmids further exacerbates resistance in hvKp [33–35]. Carbapenemase-producing hvKp strains, particularly those harboring *K. pneumonia* carbapenemase (KPC), are increasingly reported in Asia [30, 33, 36–40]. China has documented a high prevalence of CR-hvKp and CR-cKp, exhibiting both hypervirulence and XDR [41]. Fatal cases of ventilator-associated pneumonia caused by CR-hvKp have been reported in China, underscoring the urgent need for containment measures in regions where CR-hvKp has been detected [33, 41]. Additionally, hvKp demonstrates resistance to aminoglycosides, trimethoprim–sulfamethoxazole, tetracyclines, and fluoroquinolones, mediated by conjugative plasmids carrying extended-spectrum *β*-lactamase (ESBL) and carbapenemase genes [11].

### 4.1. Comparison With Previously Reported Studies

Several studies have reported the emergence of ESBL-producing hvKp [42–44], with MDR-hvKp arising through two primary mechanisms: (1) horizontal gene transfer, where hvKp strains acquire antimicrobial resistance (AMR) genes or plasmids, resulting in MDR-hvKp Type I, and (2) integration of a pLVPK-like virulence plasmid into cKp, leading to MDR-hvKp Type II. Notably, MDR-hvKp type II exhibits enhanced capsule and siderophore production compared to Type I, attributed to the presence of *rmpA*/*rmpA2*, *iut*, and *iro* genes [45]. There is ongoing debate regarding the necessity of stringent precautions when managing ESBL-producing MDR-hvKp. Nevertheless, contact precautions, screening of MDR-hvKp carriers, and active surveillance remain critical [46, 47]. All *K. pneumoniae* strains, including hvKp, demonstrate intrinsic resistance to ampicillin and ticarcillin but remain susceptible to nitrofurantoin [11]. In the present study, CR was detected in 18.07% of *K. pneumoniae* isolates, comprising 2.4% hvKp and 15.66% cKp. Among hvKp strains, 14.28% were CR-hvKp, whereas CR-cKp prevalence was 18.84%. A 2022 study by Pourgholi et al. in Tehran reported an 8.32% prevalence of CR-Kp, with the most common carbapenemase genes being *bla*_VIM_ (39.4%), *bla*_*I*MP_ (26.8%), *bla*_NPM_ (18.3%), and *bla*_KPC_ (7.0%), respectively [48].

Similarly, Nobari et al. [49] documented resistance rates of 23.3% (meropenem), 16.1% (ertapenem), and 7.7% (imipenem) in Tehran, with the highest CR prevalence (66.6%) in urine samples, all of which were MDR. Abbasi et al. [50] in central Iran observed the highest resistance to ampicillin (98.3%), cotrimoxazole (64.4%), and cephalosporins (63.6%), while tigecycline (98.4%), colistin (96.7%), and fosfomycin (95.9%) were the most effective antibiotics. CR-Kp prevalence was 42.1%, all of which were MDR. In our study, *K. pneumoniae* strains exhibited the highest susceptibility to amikacin (78.31%) and imipenem (84.33%), while resistance was most pronounced against cotrimoxazole and cephalosporins.

HvKp virulence genes are predominantly plasmid-encoded [26]. The T6SS is a critical virulence determinant, comprising structural components (e.g., Hcp, VgrG) and energy-providing elements (e.g., IcmF) for substrate translocation [14]. Mohamed et al. demonstrated a significant association between T6SS presence and resistance to meropenem, ciprofloxacin, and levofloxacin. Additionally, all biofilm-producing strains harbored T6SS, though no correlation was found between T6SS and capsule production [14]. Liao et al. reported that T6SS-positive *K. pneumoniae* exhibited elevated resistance to piperacillin-tazobactam, ciprofloxacin, levofloxacin, and meropenem, alongside higher prevalence of resistance genes *bla*_NDM_, *bla*_SHV_, *bla*_CTXM-14_, *rmtB*, *qnrS*, and *bla*_KPC_ [16]. In contrast, our study found only *bla_KPC-1_* was marginally more frequent in T6SS-positive strains, though not statistically significant. Liao et al. also noted that T6SS-positive strains carried higher levels of virulence genes (*rmpA*, *ybtS*, *kfu*, *entB*, and *fimH*), with nonsignificant increases in *mrkD*, *iutA*, and *rmpA* [16].

Zhang et al. isolated *K. pneumoniae* from bloodstream infections, identifying 16.8% as T6SS-positive, with higher prevalence among hvKp. T6SS-negative strains displayed greater antibiotic resistance, whereas T6SS-positive strains exhibited elevated virulence gene expression (*rmpA*, *rmpA2*, *aerobactin*, and *iroB*) and K1 capsular serotype prevalence [51]. Similarly, another study reported 20.1% T6SS prevalence in *K. pneumoniae* bloodstream infections, with T6SS-positive strains showing higher K1 serotype and virulence gene carriage. Again, T6SS-negative strains demonstrated greater antibiotic resistance [20]. Liu et al. found 36.1% T6SS positivity in abscess-derived *K. pneumoniae*, with T6SS-positive strains harboring more virulence genes (*rmpA*, *rmpA2*, *aerobactin*, *iroB*, *wcaG*, and *kfu*) and K1 serotype. T6SS-negative strains exhibited broader resistance, except to cefazolin and tigecycline [5].

In our study, T6SS-positive strains showed higher (but nonsignificant) prevalence of *mrkD, iroB, iutA, iucA*, and *peg-344*, whereas *rmpA* was more frequent in T6SS-negative strains. A significant correlation was observed only for aztreonam (*p* = 0.0116) and tetracycline (*p* = 0.0116) resistance. T6SS-negative strains showed higher resistance to aztreonam, tetracycline, amikacin, chloramphenicol, ceftriaxone, and cefotaxime compared to T6SS-positive strains; however, this difference was not statistically significant. Further research is needed to clarify the association between T6SS and antibiotic resistance, particularly across different infection sources. *K. pneumoniae* spreads in hospitals through three main routes: (1) contaminated hospital environments, (2) direct person-to-person contact, and (3) hvKp transmission. Unlike typical hospital-associated strains, hvKp is usually antibiotic-sensitive and primarily community-acquired. Current evidence suggests the human intestine is its main reservoir, though the original environmental source remains unidentified [52–54].

## 5. Conclusion

One of the most pressing challenges in modern medicine is the increasing prevalence of antibiotic-resistant bacterial strains. *K. pneumoniae*, a common nosocomial pathogen, exemplifies this issue. In the present study, *K. pneumoniae* isolates from western Iran exhibited high resistance to multiple antibacterial agents. Notably, the presence of the T6SS did not show a significant correlation with resistance genes or specific virulence factors. While previous studies have reported associations between T6SS and antimicrobial resistance or hypervirulence, our findings suggest potential strain-specific or regional variations, warranting further investigation.

### 5.1. Implications and Future Research Directions

All isolates (100%) were MDR, with 18.07% demonstrating CR, highlighting a serious public health concern, particularly in clinical settings with limited therapeutic options. A statistically significant association was observed between T6SS-positive isolates and ciprofloxacin resistance (*p* = 0.0219), suggesting a potential role of T6SS in fluoroquinolone resistance. However, the underlying mechanism remains unclear and requires further elucidation. Contrary to prior studies linking T6SS to hypervirulence, no significant differences in virulence or resistance gene profiles were detected between T6SS-positive and T6SS-negative isolates, indicating possible geographical or genetic variability in T6SS functionality. Additionally, the similar antibiotic resistance patterns observed in hv (hvKp) and cKp strains—except for ciprofloxacin, tetracycline, and azithromycin—suggest convergent evolution in resistance mechanisms, complicating targeted treatment approaches. Mechanistic studies should investigate the molecular pathways by which T6SS contributes to ciprofloxacin resistance, utilizing in vitro competition assays (e.g., against *E. coli*) alongside transcriptomic analysis to identify T6SS-regulated genes involved in antibiotic efflux or target modification. Additionally, given the global emergence of CR-hvKp strains associated with severe, treatment-resistant infections, enhanced surveillance in Iran is essential to monitor their prevalence and clinical impact. This should include tracking plasmid-mediated horizontal gene transfer of virulence and resistance determinants to evaluate dissemination risks and inform containment strategies.

Employ *in vivo* models (e.g., *Galleria mellonella* or murine infection) to assess virulence and evaluate therapeutic strategies:

○ Investigate the contribution of T6SS to virulence in hvKp versus cKp.
○ Analyze biofilm formation and serum resistance in T6SS-positive isolates.
○ Explore antivirulence agents targeting T6SS assembly or effector proteins.
○ Test combination therapies (e.g., *β*-lactam/*β*-lactamase inhibitors) for MDR isolates, particularly CR strains.

### 5.2. Geographic Variability Analysis

Compare T6SS prevalence, resistance patterns, and virulence gene profiles across regions (e.g., Iran vs. China/Kenya) to identify location-specific risk factors.

### 5.3. Summary

This study highlights the urgent need for enhanced surveillance of MDR *K. pneumoniae*, particularly given the unexpected association between the T6SS and ciprofloxacin resistance. Future research should prioritize three key areas: first, elucidating the mechanistic basis of T6SS-mediated antibiotic resistance; second, strengthening surveillance programs to monitor the emergence and spread of CR-hvKp strains; and third, developing novel therapeutic approaches to simultaneously address the dual challenges of heightened virulence and antimicrobial resistance that characterize these difficult-to-treat pathogens.

## Figures and Tables

**Table 1:** Primer sequences that were used in this study.

**Virulence factor genes**	**Primer sequence (5**⁣′**→3**⁣′**)**	**Amplicon size (bp)**	**Annealing temperature (°C)**	**Reference**
*mrkD*	F: AAGCTATCGCTGTACTTCCGGCA	340	64	[20]
	R: GGCGTTGGCGCTCAGATAGG			
*iroB*	F: GTGTTGGATTCCGCCAGTGA	366	61	[21]
	R: TTCCGCCGCTACCTCTTCA			
*peg-344*	F: GCGGGAAAGGACAGAAAGCCAGTG	332	56	[21]
	R: GAGGGAAGATGAGAAATACGAGC			
*iucA*	F: AATCAATGGCTATTCCCGCTG	239	62	[22]
	R: CGCTTCACTTCTTTCACTGACAGG			
*iutA*	F: GCCGCTAGGTTGGTGATGT	949	61	[21]
	R: CTCTGGTCGTGCTGGTTGA			
*rmpA*	F: GAGTATTGGTTGACAGCAGGATR:	250	53	[21]
	AGCCGTGGATAATGGTTTAC			
*hcp,*	F: TCCCGACCGATAACAACAACACC	242	42	[20]
	R: GATGTCGTGCATCAGGGGAT			
*vgrG*	F: TGAGCGTGTTTGTGCGAAAG	259	42	[20]
	R: TGACGCCCGTAATATCCTGC			
*icmF*	F: GACCGCTTACGGACAACTGAR:	485		[20]
	CACTCAGCACCCAGTCCATT			
*bla*_KPC-1_	F: CGTCTAGTTCTGCTGTCTTG	798	55	[23]
	R:CTTGTCATCCTTGTTAGGCG			
*bla*_OXA-48_	F-GCGTGGTTAAGGATGAACAC	745	60	[24]
	R-CATCAAGTTCAACCCAACCG			
*bla*_NDM-1_	F: GGTTTGGCGATCTGGTTTTC	621	54	[23]
	R: CGGAATGGCTCATCACGATC			

**Table 2:** Demographic data, prevalence of virulence, *β*-lactamases, and T6SS genes and susceptibility pattern in *K. pneumoniae* isolates.

**ID**	**Strain characterizations**				**Virulence factor genes**						***β*-Lactamases genes**				**T6SS**		
	**Ward**	**Sample type**	**String test**	**Tellurite resistance**	***mrkD***	***iroB***	***peg-344***	***iucA***	***iutA***	***rmpA***	***bla***_**KPC-1**_	***bla***_**OXA-48**_	***bla***_**NDM-1**_	***bla***_**VIM**_	***hcp***	***vgrG***	***icmF***
1	ICU	Tracheal aspiration	−	−	−	−	+	+	−	−	+	−	−	−	−	−	−
2	ICU	Tracheal aspiration	+	−	−	−	−	+	+	−	+	+	−	−	−	−	+
3	ICU	Blood	−	+	−	−	−	−	−	−	+	−	+	−	−	−	−
4	ICU	Pleural fluid	−	−	−	−	−	+	+	−	+	−	−	−	−	−	−
5	ICU	Tracheal aspiration	+	+	−	−	−	−	−	−	−	−	−	−	−	+	−
6	Infectious diseases	Urine	−	−	+	−	−	−	−	−	−	−	−	−	−	−	−
7	Emergency	Pleural fluid	−	−	−	−	−	−	−	+	−	−	−	−	−	−	−
8	ICU	Pleural fluid	+	−	−	−	−	−	−	−	−	+	−	−	−	−	−
9	Emergency	Urine	−	+	−	+	+	−	−	−	−	−	−	−	+	−	+
10	ICU	Pleural fluid	−	+	−	+	−	−	−	−	+	−	−	−	−	+	−
11	Infectious diseases	Urine	−	−	−	−	−	+	−	−	+	−	−	−	−	−	−
12	Nephrology	Urinary catheter	−	−	−	−	−	−	−	−	+	−	−	−	+	−	−
13	ICU	Urine	+	−	−	−	−	−	−	−	+	−	+	−	−	−	−
14	Emergency	Urine	−	+	−	−	−	+	−	−	+	+	−	−	−	+	−
15	Emergency	Tracheal aspiration	−	−	+	−	−	−	−	+	+	−	−	−	−	−	−
16	Emergency	Urine	−	−	−	−	−	−	−	−	+	−	−	−	−	−	−
17	ICU	Tracheal aspiration	+	+	−	−	+	+	−	−	+	−	−	−	−	−	+
18	Infectious diseases	Urine	−	−	−	−	−	−	−	−	−	−	−	−	−	−	−
19	ICU	Tracheal aspiration	−	−	−	−	−	−	−	−	−	+	−	−	−	+	−
20	Emergency	Tracheal aspiration	−	−	−	−	+	−	−	−	−	−	−	−	−	−	−
21	Neurology	Blood	+	−	−	−	+	−	−	−	−	−	−	−	−	−	−
22	ICU	Pleural fluid	−	−	−	−	−	+	−	−	−	−	−	−	−	−	−
23	Pediatrics	Urine	+	+	−	−	+	−	−	−	+	−	−	−	+	+	−
24	ICU	Tracheal aspiration	−	−	−	−	−	−	−	−	+	−	−	−	−	−	−
25	Surgery	Urine	−	+	−	−	−	+	−	−	+	−	+	−	−	−	+
26	ICU	Urine	−	−	+	−	+	−	−	−	−	−	+	−	−	+	−
27	ICU	Tracheal aspiration	−	+	+	−	−	−	−	−	−	−	−	−	−	−	−
28	Surgery	Urine	−	−	−	−	−	−	−	−	−	+	−	−	−	−	−
29	Emergency	Tracheal aspiration	+	+	−	−	−	−	−	−	−	−	−	−	−		−
30	Nephrology	Urine	−	−	−	+	−	−	−	−	−	−	−	−	−	−	+
31	ICU	Urinary catheter	−	+	−	−	−	−	−	+	−	−	−	−	−	−	−
32	ICU	Blood	−	−	−	−	−	−	−	+	−	−	−	−	−	−	−
33	Nephrology	Urine	−	+	−	−	−	−	−	+	−	−	−	−	−	−	−
34	ICU	Tracheal aspiration	−	−	−	−	−	−	−	−	+	−	−	−	+	−	−
35	Emergency	Blood	+	−	−	−	−	−	−	−	−	−	+	−	−	−	−
36	Emergency	Urinary catheter	−	−	+	−	−	−	−	−	−	−	−	−	−	−	+
37	Nephrology	Urine	−	−	−	−	−	−	−	+	−	−	−	−	−	−	−
38	Surgery	Urine	−	+	−	−	−	−	−	−	−	−	−	−	−	+	−
39	ICU	Urine	−	−	−	−	−	+	−	−	−	+	−	−	−	−	−
40	Infectious diseases	Urine	+	−	−	−	−	−	−	+	−	−	−	−	−	−	−
41	ICU	Tracheal aspiration	−	−	+	−	−	−	−	+	−	−	−	−	−	−	−
42	ICU	Urine	−	−	−	−	−	−	−	−	−	−	−	−	−	−	−
43	ICU	Urine	−	+	−	−	−	−	+	−	+	−	−	−	+	−	+
44	Emergency	Urinary catheter	−	−	−	−	−	−	−	−	−	−	−	−	−	−	−
45	Infectious diseases	Urine	−	−	−	−	−	−	−	−	−	−	−	−	−	−	−
46	ICU	CSF	−	+	−	−	−	+	−	−	−	−	−	−	−	+	+
47	Surgery	Urine	−	−	−	−	−	−	−	−	−	+	−	−	−	−	−
48	Pediatrics	Urine	−	−	−	−	−	−	−	−	−	−	−	−	−	−	−
49	ICU	Tracheal aspiration	−	−	+	−	−	−	−	−	+	−	+	−	−	−	−
50	ICU	Tracheal aspiration	−	−	−	−	−	−	−	−	−	−	−	−	−	−	−
51	Emergency	Urine	+	+	−	−	−	−	−	−	−	−	−	−	+	−	−
52	ICU	Urinary catheter	−	−	−	−	−	−	−	−	−	+	−	−	−	−	−
53	Infectious diseases	Tracheal aspiration	−	−	−	−	−	−	−	−	−	−	−	−	−	−	−
54	Infectious diseases	Urine	−	−	+	−	−	−	−	−	+	−	−	−	−	−	−
55	ICU	Tracheal aspiration	−	−	−	−	−	−	−	−	−	−		−	−	+	−
56	Infectious diseases	Urinary catheter	−	−	−	−	−	+	−	−	−	−	−	−	−	−	−
57	Neurology	Tracheal aspiration	+	−	−	−	−	−	−	−	−	−	−	−	−	−	+
58	Infectious diseases	Urine	−	+	−	−	−	+	−	−	−	+	−	−	+	+	−
59	ICU	Tracheal aspiration	−	−	−	−	−	−	−	−	+	−	−	−	−	−	−
60	Emergency	Urine	−	+	+	−	+	−	−	−	−	−	−	−	−	+	−
61	Emergency	Blood	−	−	−	−	+	−	−	−	−	+	−	−	−	−	−
62	Surgery	Tracheal aspiration	+	−	−	−	−	−	−	+	+	−	−	−	−	−	−
63	Neurology	Urine	−	−	−	−	−	−	−	+	−	−	−	−	−	−	−
64	Pediatrics	Blood	−	+	−	−	−	−	−	−	−	−	−	−	−	−	−
65	ICU	Tracheal aspiration	−	−	−	+	+	−	−	−	−	−	−	−	−	−	−
66	ICU	Urine	−	+	−	−	+	−	−	−	−	+	−	−	−	+	−
67	Infectious diseases	Urinary catheter	−	−	−	−	+	−	−	−	−	−	−	−	−	−	−
68	Pediatrics	Urine	+	+	−	−	+	−	−	−	+	−	−	−	−	−	+
69	Neurology	Blood	−	+	−	−	−	−	+	−	−	−	−	−	+	+	−
70	Surgery	Urine	−	−	−	−	−	−	+	−	−	−	−	−	−	−	−
71	Surgery	Urinary catheter	+	−	−	−	−	−	−	−	+	−	+	−	−	−	−
72	Nephrology	Urinary catheter	−	+	−	−	−	−	−	−	−	−	−	−	−	−	−
73	Infectious diseases	Urinary catheter	+	+	−	−	−	−	−	−	−	+	−	−		−	−
74	Emergency	Urine	−	−	−	−	−	−	−	−	−	−	−	−	−	−	−
75	Neurology	Tracheal aspiration	+	−	−	−	−	−	−	−	−	+	−	−	−	−	−
76	Infectious diseases	Urine	−	−	−	−	−	−	−	−	+	−	−	−	−	−	−
77	Infectious diseases	Urine	+	−	−	−	−	+	−	+	−	−	−	−	−	−	−
78	Emergency	Blood	−	+	+	−	−	−	+	−	−	−	−	−	−	+	−
79	ICU	Urine	−	−	−	−	+	−	−	+	−	−	−	−	−	−	−
80	Infectious diseases	Urinary catheter	+	−	−	+	−	+	+	−	−	+	−	−	−	−	−
81	Emergency	Urine	+	−	+	−	−	−	+	−	+	−	−	−	−	−	−
82	ICU	Blood	−	+	−	−	−	+	−	−	−	−	−	−	−	+	+
83	Infectious diseases	Urine	−	−	−	−	−	−	−	−	−	−	−	−	−	−	−

**Table 3:** Antibiotic susceptibility pattern of *K. pneumoniae* isolates.

**Susceptibility pattern**	**Antibiotic resistance no (%)**												
		**CIP**	**CTX**	**CFP**	**CRO**	**CHO**	**AMK**	**GEN**	**SXT**	**TET**	**MER**	**IMI**	**AZT**
**CKp (** **n** = 69**)**	S	31 (44.92)	17 (24.63)	20 (28.98)	3 (4.34)	32 (46.37)	53 (76.81)	44 (63.76)	27 (39.13)	37 (53.62)	53 (76.81)	59 (85.5)	33 (47.82)
	I	0 (0)	1 (1.44)	6 (8.69)	11 (15.94)	0 (0)	5 (7.24)	2 (2.89)	1 (1.44)	2 (2.89)	3 (4.34)	0 (0)	0 (0)
	R	38 (55.07)	51 (73.91)	43 (62.31)	55 (79.71)	37 (53.62)	11 (15.94)	23 (33.33)	41 (59.42)	30 (43.47)	13 (18.84)	10 (14.49)	36 (52.17)

**HvKp (** **n** = 14**)**	S	5 (35.71)	4 (28.57)	2 (14.28)	0 (0)	7 (50)	12 (85.71)	10 (71.42)	3 (21.42)	11 (78.57)	12 (85.71)	11 (78.57)	5 (35.71)
	I	0 (0)	1 (7.14)	0 (0)	2 (14.28)	0 (0)	2 (14.28)	0 (0)	0 (0)	1 (7.14)	0 (0)	0 (0)	0 (0)
	R	9 (64.28)	9 (64.28)	12 (85.71)	12 (85.71)	7 (50)	0 (0)	4 (28.57)	11 (78.57)	2 (14.28)	2 (14.28)	3 (21.42)	9 (64.28)

**Total *Klebsiella* isolates (** **n** = 83**)**	S	36 (43.37)	21 (25.3)	22 (26.5)	3 (3.61)	39 (46.98)	65 (78.31)	54 (65.06)	30 (36.14)	48 (57.83)	65 (78.31)	70 (84.33)	38 (45.78)
	I	0 (0)	2 (2.4)	6 (7.22)	13 (15.66)	0 (0)	7 (8.43)	2 (2.4)	1 (1.2)	3 (3.61)	3 (3.61)	0 (0)	0 (0)
	R	47 (56.62)	60 (72.28)	55 (66.26)	67 (80.72)	44 (53.01)	11 (13.25)	27 (32.53)	52 (62.65)	32 (38.55)	15 (18.07)	13 (15.66)	45 (54.21)

**Table 4:** Antimicrobial resistance pattern of hvKp and cKp isolates.

**Antimicrobial agent**	**CKp (** **n** = 69**)**		**HvKp (** **n** = 14**)**		**Total *K. pneumoniae* isolates (** **n** = 83**) N (%)**	**p** ** value**
	**Positive**	**Negative**	**Positive**	**Negative**		
CIP	38 (55.07)	31 (44.92)	9 (64.28)	5 (35.71)	47 (56.62)	0.0347
CTX	51 (73.91)	18 (26.08)	9 (64.28)	5 (35.71)	60 (72.28)	0.3089
CFP	43 (62.31)	26 (37.68)	12 (85.71)	2 (14.28)	55 (66.26)	0.0806
CRO	55 (79.71)	14 (20.28)	12 (85.71)	2 (14.28)	67 (80.72)	0.3267
CHO	37 (53.62)	32 (46.37)	7 (50)	7 (50)	44 (53.01)	0.0577
AMK	11 (15.94)	58 (84.05)	0 (0)	14 (100)	11 (13.25)	0.3446
GEN	23 (33.33)	43 (62.31)	4 (28.57)	10 (71.42)	27 (32.53)	0.1674
SXT	41 (59.42)	28 (40.57)	11 (78.57)	3 (21.42)	52 (38.55)	0.0577
TET	30 (43.47)	39 (56.52)	2 (14.28)	12 (85.71)	32 (38.55)	0.0116
MER	13 (18.84)	56 (81.15)	2 (14.28)	12 (85.71)	15 (18.07)	0.344
IMI	10 (14.49)	59 (85.5)	3 (21.42)	11 (78.57)	13 (15.66)	0.4078
AZT	36 (52.17)	33 (47.82)	9 (64.28)	5 (35.71)	45 (54.21)	0.0116

**Table 5:** Antimicrobial resistance of T6SS-positive and T6SS-negative *K. pneumoniae* isolates.

**Antimicrobial resistance**	**T6SS-negative (** **n** = 56**)**		**T6SS-positive (** **n** = 27**)**		**All** **N** = 83** (100)**	**p** ** value**
	**Positive**	**Negative**	**Positive**	**Negative**		
CIP	31 (55.35)	25(44.64)	16 (59.25)	11 (40.7)	47 (56.62)	0.0219
CTX	41 (73.21)	15(26.78)	19 (70.37)	8(29.69)	60 (72.28)	0.3039
CFP	34 (60.71)	22(39.28)	21 (77.770	6 (22.22)	55 (66.26)	0.0656
CRO	47 (83.92)	9(16.07)	20 (74.07)	7(25.92)	67 (80.72)	0.4529
CHO	31 (55.35)	25(44.64)	13 (48.14)	14 (51.28)	44 (53.01)	0.1508
AMK	9 (16.07)	47 (83.9)	2 (7.4)	25 (92.59)	11 (13.25)	0.3039
GEN	18 (32.14)	38(58.92)	9 (33.33)	18 (66.66)	27 (32.53)	0.2308
SXT	32 (57.14)	24 (42.85)	20 (74.07)	7(25.92)	52 (38.55)	0.1087
TET	23 (40.07)	33 (58.92)	9 (33.33)	18(66.66)	32 (38.55)	0.4576
MER	10 (17.85)	46(82.14)	5 (18.51)	22(81.48)	15 (18.07)	0.3692
IMI	6 (10.71)	50 (89.28)	7 (25.92)	20 (74.07)	13 (15.66)	0.5212
AZT	31 (55.35)	25 (44.64)	14 (51.85)	13(48.14)	45 (54.21)	0.1087

**Table 6:** Virulence factor and *β*-lactamase genes between T6SS-negative and T6SS-positive *K. pneumoniae* isolates.

**Virulence factor genes**	**Total (** **n** = 83**)**		**T6SS-negative (** **n** = 56**)**		**T6SS-positive (** **n** = 27**)**		**p** ** value**
	**Positive**	**Negative**	**Positive**	**Negative**	**Positive**	**Negative**	
*mrkD*	11 (13.25)	72 (86.74)	7 (12.5)	49 (87.5)	4 (14.81)	23 (85.18)	0.4269
*iroB*	5 (6.02)	78 (93.97)	2 (3.57)	54 (96.42)	3 (11.11)	24 (88.88)	0.5212
*peg-344*	14 (16.86)	69 (83.13)	7 (12.5)	49 (87.5)	7 (25.92)	20 (74.07)	0.5
*iucA*	15 (18.07)	68 (81.92)	7 (12.5)	49 (87.5)	8 (29.62)	19 (70.37)	0.5212
*iutA*	8 (9.63)	75 (90.36)	4 (7.14)	52 (92.85)	4 (14.81)	23 (85.18)	0.5
*rmpA*	12 (14.45)	71 (85.54)	12 (21.42)	44 (78.57)	0 (0.0)	27 (100)	0.1087
*β-lactamase genes*							
*bla*_KPC-1_	25 (30.12)	58 (69.87)	16 (28.57)	40 (71.42)	9 (33.33)	18 (66.66)	0.3039
*bla*_OXA-48_	14 (16.86)	69 (83.13)	9 (16.07)	47 (83.92)	5 (15.51)	22 (81.48)	0.393
*bla*_NDM-1_	7 (8.43)	76 (91.56)	5 (8.92)	51 (91.07)	2 (7.4)	25 (92.59)	0.4269
hvkp	14 (16.86)	69 (83.13)	0 (0.0)	56 (100)	14 (51.85)	13 (48.14)	0.7004

*Note: bla*_VIM_ gene not detected.

**Table 7:** Virulence factor and *β*-lactamase genes between hvKp and cKp isolates.

**Virulence factor genes**	**Total (** **N** = 83**)**		**HvKp (** **n** = 14**) N (%)**		**CKp (** **n** = 69**) N (%)**		**p** ** value**
	Positive	Negative	Positive	Negative	Positive	Negative	

*mrkD*	11 (13.25)	72 (86.74)	2(14.28)	12 (85.71)	9(13.04)	60 (86.95)	0.407
*iroB*	5 (6.02)	78 (93.97)	1(7.14)	13 (92.85)	4(5.79)	65 (94.2)	0.463
*peg-344*	14 (16.86)	69 (83.13)	6(42.85)	8 (57.14)	8(11.59)	61 (88.4)	0.476
*iucA*	15 (18.07)	68 (81.92)	6(42.85)	8 (57.14)	9(13.04)	60 (86.95)	0.463
*iutA*	8 (9.63)	75 (90.36)	3(21.42)	11 (78.57)	5(7.24)	64 (92.75)	0.476
*rmpA*	12 (14.45)	71 (85.54)	0(0.0)	14 (100)	12(17.39)	57 (82.6)	0.326

***β*-Lactamase genes**							
	Positive	Negative	Positive	Negative	Positive	Negative	

*bla*_KPC-1_	25 (30.12)	58 (69.87)	6(42.85)	8 (57.14)	19(27.53)	50 (72.46)	0.308
*bla*_OXA-48_	14 (16.86)	69 (83.13)	3(21.42)	11 (78.57)	11(15.94)	58 (84.05)	0.392
*bla*_NDM-1_	7 (8.43)	76 (91.56)	1(7.14)	13 (92.85)	6(8.69)	63 (91.3)	0.436

## Data Availability

The data that support the findings of this study are available on request from the corresponding authors.

## Nomenclature

CR: carbapenem-resistant
CSF: cerebrospinal fluid
cKp: classic *K. pneumoniae*
Hcp: hemolysin co-regulatory protein
HMV: hypermucoviscous
hvKp: hypervirulent *K. pneumoniae*
LIA: lysine iron agar
MDR: multidrug-resistant
MHA: Mueller–Hinton agar
PCR: polymerase chain reaction
TSI: triple sugar iron
T6SS: Type VI secretion system
UTI: urinary tract infections
VgrG: valine-glycine repeat protein G
